# Mechanism of Atg9 recruitment by Atg11 in the cytoplasm-to-vacuole targeting pathway

**DOI:** 10.1016/j.jbc.2022.101573

**Published:** 2022-01-08

**Authors:** Nicolas Coudevylle, Bartłomiej Banaś, Verena Baumann, Martina Schuschnig, Anna Zawadzka-Kazimierczuk, Wiktor Koźmiński, Sascha Martens

**Affiliations:** 1 Max Perutz Laboratories, University of Vienna, Vienna, Austria; 2 Faculty of Chemistry, Biological and Chemical Research Centre, University of Warsaw, Warsaw, Poland; 3 Vienna BioCenter PhD Program, Doctoral School of the University of Vienna and Medical University of Vienna, Vienna, Austria

**Keywords:** nuclear magnetic resonance, intrinsically disordered proteins, autophagy, isothermal titration calorimetry, yeast metabolism, Ape1, aminopeptidase 1, Cvt, cytoplasm-to-vacuole targeting, ER, endoplasmic reticulum, GFP, green fluorescent protein, HSQC, heteronuclear single-quantum coherence, IDP, intrinsically disordered protein, ITC, isothermal titration calorimetry, MBPPI, microscopy-based protein–protein interaction, NMR, nuclear magnetic resonance, NTD, N-terminal domain, SMFT, sparse multidimensional Fourier transform

## Abstract

Autophagy is a lysosomal degradation pathway for the removal of damaged and superfluous cytoplasmic material. This is achieved by the sequestration of this cargo material within double-membrane vesicles termed autophagosomes. Autophagosome formation is mediated by the conserved autophagy machinery. In selective autophagy, this machinery including the transmembrane protein Atg9 is recruited to specific cargo material *via* cargo receptors and the Atg11/FIP200 scaffold protein. The molecular details of the interaction between Atg11 and Atg9 are unclear, and it is still unknown how the recruitment of Atg9 is regulated. Here we employ NMR spectroscopy of the N-terminal disordered domain of Atg9 (Atg9-NTD) to map its interaction with Atg11 revealing that it involves two short peptides both containing a PLF motif. We show that the Atg9-NTD binds to Atg11 with an affinity of about 1 μM and that both PLF motifs contribute to the interaction. Mutation of the PLF motifs abolishes the interaction of the Atg9-NTD with Atg11, reduces the recruitment of Atg9 to the precursor aminopeptidase 1 (prApe1) cargo, and blocks prApe1 transport into the vacuole by the selective autophagy-like cytoplasm-to-vacuole (Cvt) targeting pathway while not affecting bulk autophagy. Our results provide mechanistic insights into the interaction of the Atg11 scaffold with the Atg9 transmembrane protein in selective autophagy and suggest a model where only clustered Atg11 when bound to the prApe1 cargo is able to efficiently recruit Atg9 vesicles.

Autophagy is a conserved pathway for the delivery of cytoplasmic material into the lysosomal system for degradation. The material referred to as cargo is encapsulated within double-membrane vesicles, the autophagosomes. Upon induction of autophagy, either by the lack of nutrients or, in selective autophagy, by the presence of the cargo autophagosome biogenesis is initiated (1). Autophagosome formation progresses through the assembly of a small membrane structure termed phagophore (or isolation membrane), which gradually engulfs the cargo as it expands. Subsequently, a scission reaction mediates the closure of the phagophore resulting in the formation of an autophagosome. Finally, the outer membrane of the autophagosome fuses with the vacuole in yeast (or lysosome in mammals), wherein the inner membrane and the cargo are degraded.

Autophagosome biogenesis is mediated by a conserved set of functional modules forming the autophagy machinery. These modules are the Atg1 kinase complex, Atg9 vesicles, the class III phosphatidylinositol 3-phosphate kinase complex 1 (PI3KC3-C1), the Atg2-Atg18 lipid binding and transfer complex, Atg21 and the Atg8 lipidation machinery including the Atg12–Atg5-Atg16 complex (the nomenclature refers to the *Saccharomyces cerevisiae* proteins) (2, 3, 4, 5). In selective autophagy, this machinery is recruited to the cargo material through cargo receptors, which first recruit the Atg11 scaffold protein to the cargo (6, 7, 8, 9). The 135 kDa Atg11 forms homodimers that consist of four coiled-coil domains and a so-called “claw-domain” that specifically interacts with phosphorylated cargo receptors (7, 10, 11, 12, 13, 14, 15, 16). Atg11 in turn recruits the Atg1 kinase complex and Atg9 (6, 7, 11, 17, 18, 19).

Atg9 consists of a four-helices transmembrane core flanked by two large disordered and cytosolic termini (Fig. 1, *A* and *B*) (20, 21, 22). It forms trimers that localize to trans-Golgi-derived vesicles, a few of which are recruited to the site of autophagosome formation (23, 24, 25). Once recruited to the cargo, Atg9 vesicles function as seeds to initiate autophagosome formation (6, 23). Through its recently described scramblase activity, Atg9 allows phagophore elongation by distributing the ER-derived lipids transported by Atg2 between the two leaflets of the phagophore membrane (5, 6, 20, 21, 26, 27, 28, 29, 30). The recruitment of Atg9 to the site of autophagosome formation by Atg11 is therefore a crucial event in autophagosome biogenesis.

**Figure 1:**
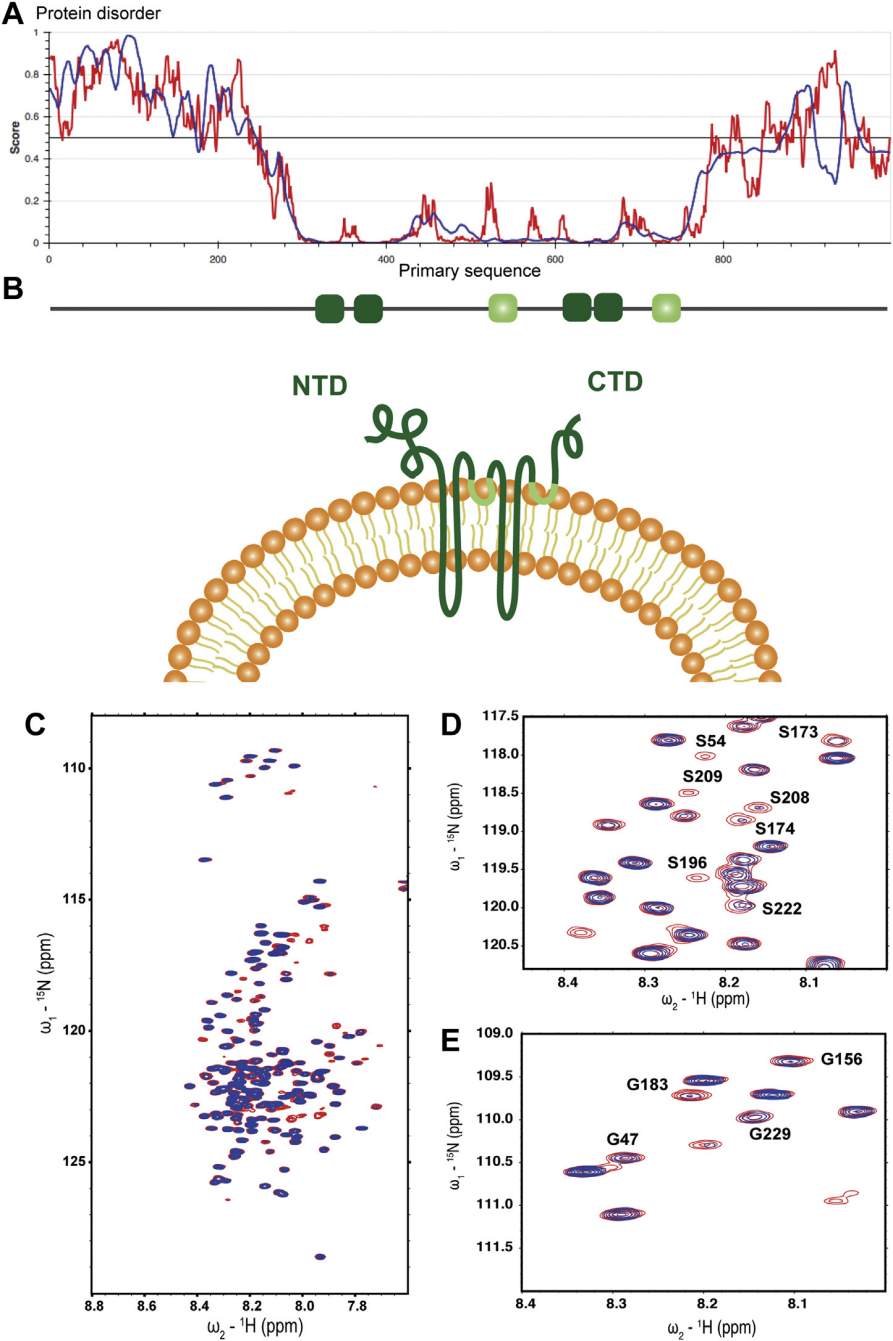
**Atg9-NTD is disordered in solution and interacts with Atg11.***A*, predicted disorder score along the primary sequence of Atg9 according to the IUPred (*red trace*) and ANCHOR (*blue trace*) algorithms. *B*, schemes of Atg9. Transmembrane (TM) domains are represented as plain *dark green bars*, partially inserted helical segments as *light green bars*. *C*, overlay of the ^1^H-^15^N HSQC spectra of Atg9-NTD(1–285) in its free (*red resonances*) and of Atg11-bound (*blue resonances*) forms. The concentration of Atg9-NTD is 0.2 mM, the concentration of Atg11 in the bound sample is 0.04 mM. *D*, close-up on the Serine region of the HSQC spectra. *E*, close-up on the Glycine region of the HSQC spectra.

Some features of the interaction between Atg11 and Atg9 are already known. Atg11 was shown to bind to the intrinsically disordered N-terminal domain (NTD) of Atg9, and this interaction requires the coiled-coil domain 2 (CC2, residues 536–576) of Atg11 (6, 7, 19). However, the molecular details of this interaction as well as the spatiotemporal regulation of the recruitment of Atg9 vesicles to the cargo are still unclear. Here we show that the Atg9-NTD binds to Atg11 with an affinity of about 1 μM *via* two PLF motifs. Therefore, a productive high avidity interaction between Atg9 and Atg11 can only happen when Atg11 is clustered on the cargo, providing a mechanism for spatiotemporal regulation of the recruitment of Atg9 vesicles to the cargo site.

## Results

### Mapping of the Atg9-NTD–Atg11 interaction by NMR

**Figure 2:**
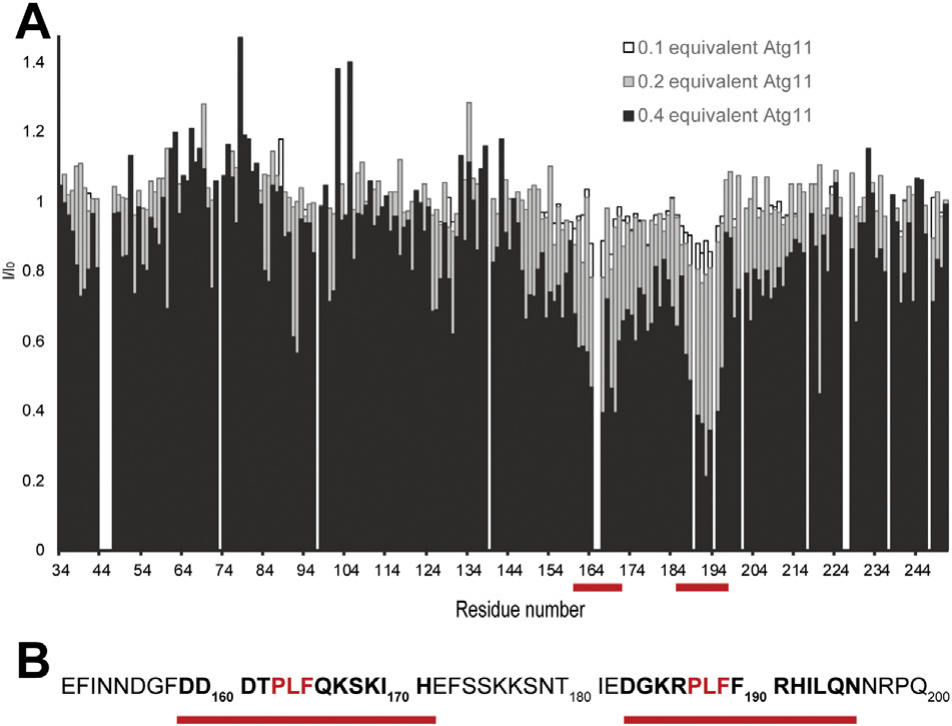
**Atg9-NTD interacts with Atg11 through two short segments.***A*, intensity ratio of the ^1^H-^15^N HSQC resonances of Atg9-NTD(29–255) in the presence of increasing amount of unlabeled Atg11, the interacting regions in Atg9-NTD(29–255) are identified by *red bars*. The concentration of Atg9-NTD(29–255) was 0.2 mM, concentrations of Atg11 were 0.02, 0.04, and 0.08 mM (0.1, 0.2, and 0.4 equivalent, respectively). *B*, primary sequence of Atg9 (residues 151–200), the residues most affected by Atg11 binding are highlighted in *bold*, the two PLF motifs are colored in *red*.

In order to obtain mechanistic insights into the interaction of Atg9 with Atg11, we used nuclear magnetic resonance (NMR) spectroscopy, an ideal method to study intrinsically disordered proteins (IDPs). Using isotopically labeled samples and ^1^H-^15^N HSQC NMR, we concluded that, as predicted, the Atg9 NTD (residues 1–285) is largely disordered in solution (Fig. 1*C*). The spectrum of the protein exhibits sharp intense resonances together with the narrow chemical shift dispersion typical of IDPs. Upon addition of substoichiometric amounts of Atg11, the intensities of individual peaks decreased indicating that these residues are specifically involved in the interaction (Fig. 1, *C*–*E*). In order to identify the binding site in the Atg9-NTD for Atg11, we next performed a resonance assignment of the fragment 1 to 285. Since the resonance assignment of large IDPs (such as Atg9 NTD) is very challenging, we employed high-dimensionality techniques (5D) combined with fast pulsing techniques, nonlinear sampling, and automated assignment procedures. Following this strategy, we could assign the backbone as well as some side chain (up to Cβ and Hβ) for residues from Ser32 to Ser250 (BMRB accession number 51011). Secondary structure propensity calculation showed that this fragment (32–250) is completely devoid of secondary structures (Fig. S1). Fragments 1 to 31 and 251 to 285 are probably partially folded and/or undergoing intermediate conformational exchange. They do not seem to affect the structural dynamics of the 32 to 250 segment, as a shorter construct of Atg9-NTD (29–255) exhibits a nearly identical ^1^H-^15^N HSQC spectrum than the longer construct (Fig. S2). Based on this assignment and in order to optimize the spectral quality by limiting resonance overlap, we used this shorter fragment to perform a ^1^H-^15^N HSQC-based titration of Atg9-NTD (29–255) by Atg11 (Figs. 2*A* and S3). Due to the large size of the Atg11 dimer (270 kDa), addition of Atg11 to Atg9 NTD did not result in chemical shift changes but rather to an Atg11 concentration-dependent intensity decrease for a specific set of resonances. This titration in combination with the assignment allowed us to identify the putative binding site for Atg11 in the Atg9 NTD, which consists of two short stretches (Fig. 2*A*), both being centered on a PLF motif (Fig. 2*B*). A few unassigned resonances also seem to be affected upon Atg11 binding (Fig. S1), suggesting that part of the N- or C-terminus might also be involved in binding to Atg11.

### Two PLF motifs in the Atg9-NTD are required for the interaction with Atg11

**Figure 3:**
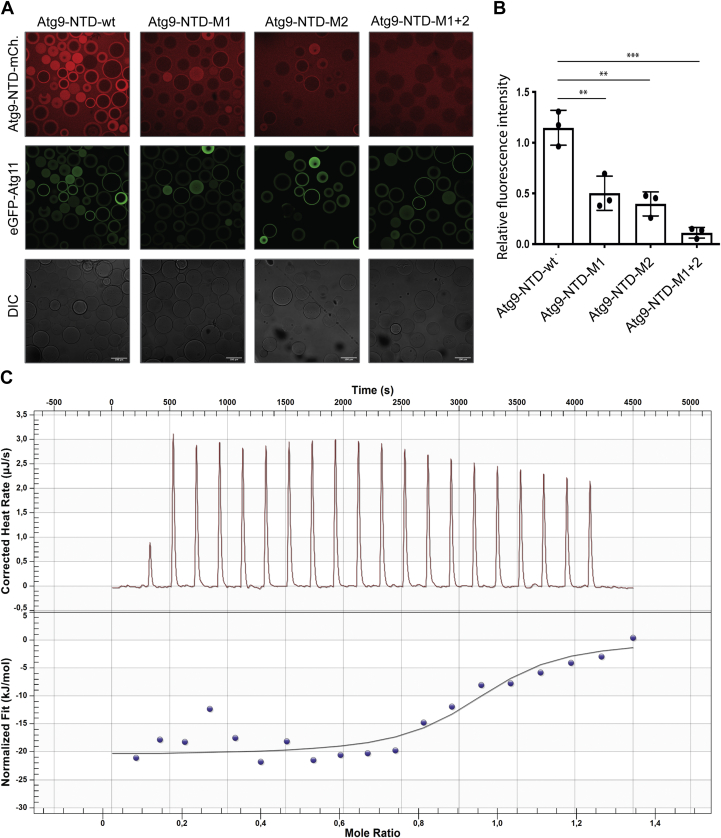
**Atg9-NTD PLF motifs are essential for the interaction with Atg11.***A*, microscopy-based protein–protein interaction assays between GFP-Trap beads coated with EGFP-Atg11 and different forms of Atg9-NTD-(1–255)-mCherry, the scale bars represent 100 μm. *B*, quantification of the mCherry signal on the beads normalized to the EGFP signal, the error bars correspond to the standard deviation between three independent replicates. *C*, isothermal titration calorimetry traces of Atg9-NTD(1–255) binding to Atg11. For data fitting, the heat of dilution of Atg11 has been subtracted from the raw data (Fig. S4). Thermodynamic parameters of binding are: *K*_*D*_ = 1.089 μM, n = 0.94, ΔH = −24.55 ± 5.6 kJ mol^−1^.

In order to test if these PLF motifs are required for the interaction of the Atg9-NTD with Atg11, we mutated the L and F of both motifs (L_164_F_165_ and L_188_F_189_) to A and tested the mutant proteins in a microscopy-based protein–protein interaction (MBPPI) assay (Fig. 3*A*). We immobilized the EGFP tagged Atg11 on GFP-Trap beads and added mCherry-tagged Atg9-NTD. As expected, we observed a robust recruitment of Atg9-NTD(1–255) to the beads coated with EGFP-Atg11 (Fig. 3, *A* and *B*). Mutation of the first PLF motif (M1) severely reduced the interaction, and mutation of the second PLF motif (M2) reduced the recruitment of the Atg9-NTD to the EGFP-Atg11-coated beads (Fig. 3, *A* and *B*) to a similar extent. Upon mutation of both motifs (M1+M2), the interaction between the Atg9-NTD and Atg11 became undetectable (Fig. 3, *A* and *B*).

Next, we characterized the interaction of the Atg9-NTD with Atg11 using ITC (Fig. 3*C*). Titration of the wild-type Atg9-NTD(1–255) into full-length Atg11 yielded a robust signal, fitting of which resulted in a K_D_ of around 1 μM and a stoichiometry of 1 (*K*_*D*_ = 1,086 ± 0,006 μM, n = 0,931 ± 0,004, Fig. S4). The affinity of the Atg9-NTD(1–255) M1 and M2 mutants for Atg11 was too low to be measured accurately by ITC.

### The two PLF motifs in the Atg9-NTD are required for the Cvt pathway

**Figure 4:**
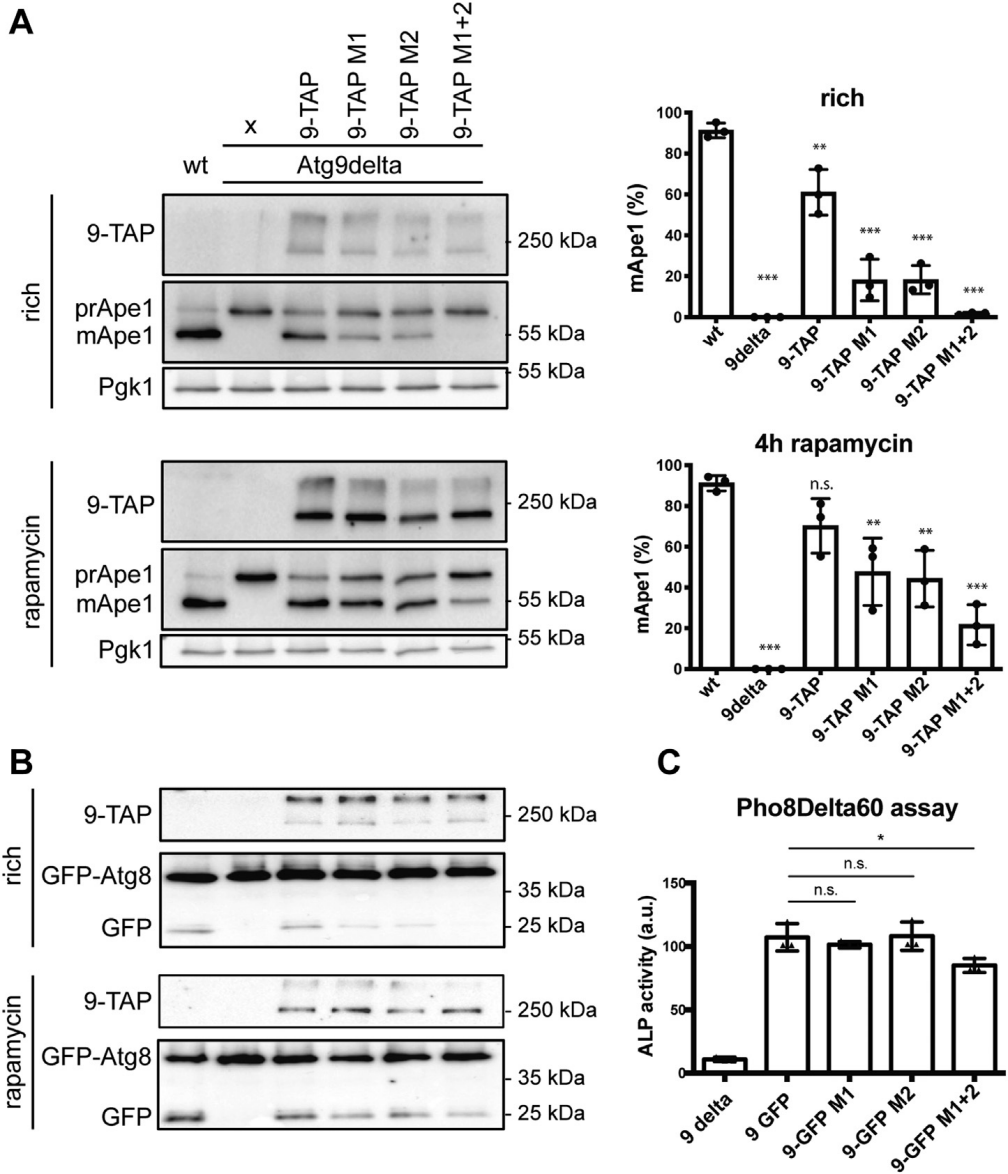
**Atg9-NTD PLF motifs are essential for selective autophagy *in vivo*.***A*, Western blot analysis of Ape1 processing of different Atg9 constructs, in rich conditions and after 4 h of treatment with rapamycin, the quantification of the Ape1 bands are shown on the right. The percentage of mApe1 is calculated in reference to total Ape1 (mApe1 + prApe1). *B*, Western blot analysis of GFP-Atg8 processing of different Atg9 constructs in rich conditions and after 4 h of treatment with rapamycin. *C*, Pho8Delta60 assay, quantification of the alkaline phosphatase activity in strains carrying different version of Atg9. For all experiments, a total of three independent replicates were conducted. The graph shows the averages, and the error bars represent the standard deviations. *p* values were calculated using Student’s *t* test. Significances are indicated with ∗ when *p* value ≤ 0.05, ∗∗ when *p* value ≤ 0.01, and ∗∗∗ when *p* value ≤ 0.001.

Having established that the two PLF motifs are *bona fide* binding sites for Atg11, at least *in vitro*, we asked what the effect of their mutation on autophagic processes in cells might be. *A priori*, we expected the PLF motifs mediating the interaction of Atg9 with Atg11 to be particularly important for selective autophagy such as the Cvt pathway, which mediates the transport of prApe1 into the vacuole wherein its propeptide is cleaved off to produce active mApe1 (mature Ape1). We therefore expressed the mutant Atg9 proteins in *Atg9*Δ cells and assessed their effects on prApe1 processing under nutrient-rich conditions by the Cvt pathway and on rapamycin-induced bulk autophagy (Fig. 4*A*). The different mutations do not appear to affect expression levels of Atg9. However, individual mutation of motif 1 and 2 resulted in reduction of prApe1 transport into the vacuole of about 70% when compared with wild-type Atg9-TAP (Fig. 4*A*). Cells expressing Atg9 with mutations in motifs 1 and 2 showed no detectable prApe1 processing under nutrient-rich conditions. In contrast, upon induction of bulk autophagy by addition of rapamycin, single mutation of motif 1 and 2 resulted in a reduction of prApe1 processing of only about 30%. prApe1 processing was also readily detectable for the double mutant with disrupted motifs 1 and 2 (Fig. 4*A*). A similar picture emerged when we determined GFP-Atg8 processing under nutrient-rich conditions and upon rapamycin-induced bulk autophagy. This assay follows the transport of GFP-Atg8 into the vacuole, wherein the Atg8 moiety is rapidly degraded while the relatively more stable GFP accumulates. As expected, under nutrient-rich conditions, the levels of free GFP were low but detectable. Individual mutation of motifs 1 and 2 resulted in a reduction of the free GFP band, while the double mutation abolished GFP-Atg8 processing completely (Fig. 4*B*). In rapamycin-induced bulk autophagy, GFP-Atg8 processing was less severely reduced and detectable for all mutants. Thus, the Cvt pathway acting under nutrient-rich conditions appears to be particularly strongly affected by the mutation of the PLF motifs in Atg9. In order to evaluate the effect of these Atg9 mutations on bulk autophagy, we also performed a Pho8Delta60 assay (31) (Fig. 4*C*). Only cells expressing Atg9 with mutations in motifs 1 and 2 exhibited a slight decrease in bulk autophagy, showing that, as expected, these mutations as well as the abolition of the Atg9/Atg11 interaction do not considerably affect bulk autophagy.

### The PLF motifs in the Atg9-NTD contribute to the recruitment of Atg9 to the prApe1 cargo through its interaction with Atg11 *in vivo*

**Figure 5:**
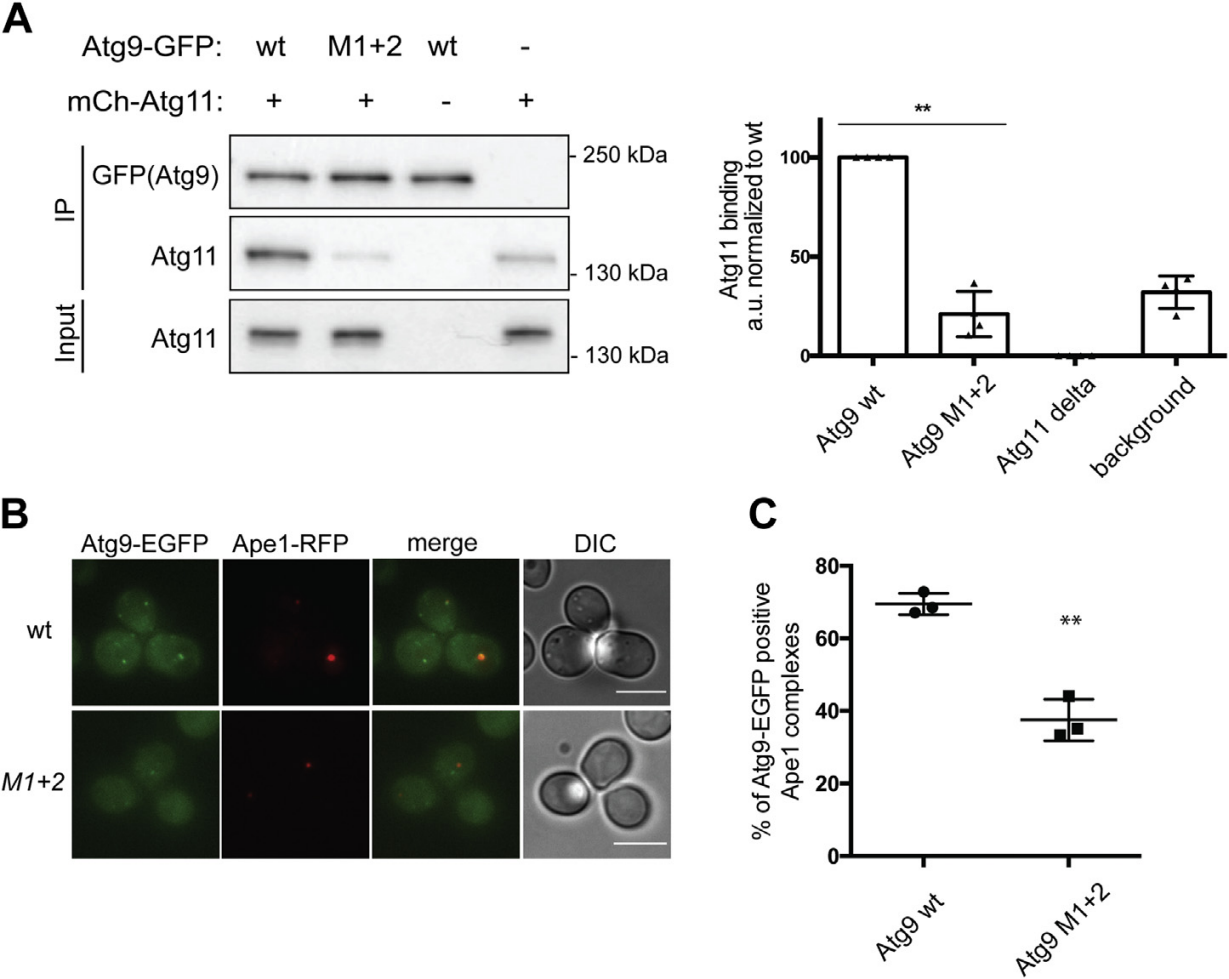
**Atg9-NTD PLF motifs are essential for the recruitment of Atg9 to the PAS through its interaction with Atg11**. *A*, Western blot analysis of Atg11 coimmunoprecipitation with different Atg9-EGFP constructs. *B*, recruitment of Atg9-EGFP and Atg9-EGFP L164A/F165A/L188A/F189A to the autophagosome formation site. Yeast cells of the indicated genotype expressing Ape1-RFP were imaged using a Deltavision ULTRA Epifluorescence microscope. *C*, percentage of Atg9-EGFP dots colocalizing with Ape1-RFP dots. In total, three independent experiments were conducted. The graph shows the averages, and the error bars represent the standard deviations. *p* values were calculated using Student’s *t* test. Scale bars: 5 μm. Significances are indicated with ∗ when *p* value ≤0.05, ∗∗ when *p* value ≤ 0.01, and ∗∗∗ when *p* value ≤ 0.001.

Finally, we asked what the basis for the block of the Cvt pathway as measured by prApe1 and GFP-Atg8 processing by mutations in the PLF motifs in the Atg9-NTD might be. Atg11 is recruited to the prApe1 cargo *via* the Atg19 cargo receptor and acts upstream of the autophagy machinery to initiate Cvt vesicle formation. We therefore expected that the loss of the Atg11–Atg9 interaction by the mutations in PLF motifs 1 and 2 results in reduced Atg9 recruitment to the prApe1 cargo. To test this hypothesis, we first checked the recruitment of Atg11 by Atg9 by coimmunoprecipitation (Fig. 5*A*). Quantification of the Atg11 Western blot signal reveals a strong binding of Atg11 to wild-type Atg9, whereas in the case of Atg9 mutated in motifs 1 and 2, the signal for Atg11 is reduced to background levels (Fig. 5*A*).

Next, we expressed wild-type and mutant Atg9-EGFP and prApe1-RFP in *Atg9*Δ cells and followed the recruitment of the two Atg9-EGFP proteins to the prApe1 cargo by fluorescence microscopy (Fig. 5*B*). Indeed, the PLF motifs 1 and 2 mutant showed a severely reduced recruitment to the prApe1 cargo suggesting that the interaction of Atg11 with the PLF motifs in the NTD of Atg9 contributes considerably to Atg9 recruitment (Fig. 5*C*).

## Discussion

It has become clear that in selective autophagy, the recruitment of the autophagy machinery is induced by the cargo *via* its recognition by a cargo receptor that in turn recruits the Atg11/FIP200 scaffold proteins (11). These scaffold proteins have a pivotal role by attracting the Atg1/ULK1 complex and Atg9 vesicles to initiate autophagosome formation *in situ*. However, the molecular details of these interactions are largely unknown as well as their mechanisms of regulation.

We recently gained insight into these processes by reconstituting *in vitro* the early steps of phagophore formation. We proposed a model in which Atg9 vesicles are recruited to the site of autophagosome biogenesis and serve as seeds for phagophore assembly by recruiting the rest of the autophagy machinery (6). Furthermore, the lipid scramblase activity of Atg9 in conjunction with Atg2 mediated transport of lipids into the phagophore and *de novo* lipid biosynthesis drive phagophore elongation (5, 6, 20, 21, 26, 27, 28, 29, 30, 32). However, in our reconstitutions (6), we used either endogenous Atg9 vesicles or reconstituted Atg9 proteoliposomes. In both cases, it is difficult to delineate the fine details of the molecular interactions and to differentiate the respective contributions of the protein and lipid parts of Atg9 vesicles.

Consequently, we focused on the soluble NTD of Atg9, which has been reported to interact with several autophagy factors, including Atg11 (19, 33, 34). Since the Atg9-NTD is predicted to be disordered, we used NMR spectroscopy to characterize the interaction between the Atg9-NTD and Atg11. Our NMR data confirm the disordered nature of the Atg9-NTD (at least for the residues 34–250). Our near-complete resonance assignment of the residues 34 to 250 of Atg9-NTD allowed us to map the binding site for Atg11. In particular, we identified two stretches of 13 and 14 residues respectively, both centered on a PLF motif. Mutation of the first or second motif to PAA resulted in a severely reduced affinity for Atg11, whereas mutation of both motifs to PAA completely abolished the interaction of the Atg9-NTD with Atg11. Our ITC measurement showed that the affinity of the Atg9-NTD for Atg11 is about 1 μM.

**Figure 6:**
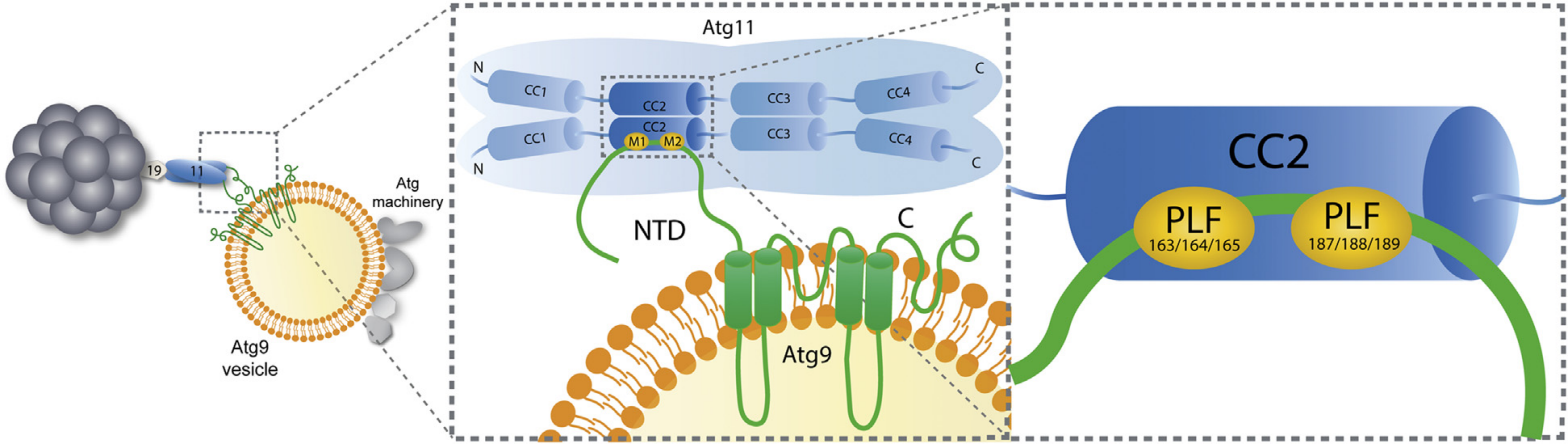
**Model of the interaction between Atg11 and Atg9 vesicles.** Atg9-NTD interacts with Atg11-CC2 through two PLF motifs. The clustering of Atg11 on prApe1 particles leads to a robust recruitment of Atg9 vesicles.

Such relatively low affinity is unlikely to result in a tight complex between Atg9 and an isolated Atg11 dimer. However, it may be sufficient to allow for an Atg9 vesicle to be robustly recruited to the cargo. Indeed, Atg11 clusters at the surface of the cargo (*via* its interaction with the cargo receptor) and will be able to form multiple interactions with several trimers of Atg9 at the surface of the vesicle, leading to a high avidity interaction between the cargo and the Atg9 vesicle (Fig. 6). In this model, the clustering of the scaffold protein to the cargo, which is regulated by the phosphorylation of the cargo receptor (35), is sufficient to lead to the recruitment of Atg9 vesicles.

Consistent with this model and the importance of the PLF motifs, we observed a reduced colocalization of prApe1 and Atg9 upon their mutation. Additionally, mutation of the PLF motifs reduced or even abolished the interaction with Atg11 *in vivo* and consequently prApe1 transport into the vacuole by the Cvt pathway. Upon induction of bulk autophagy by the addition of the Tor inhibitor rapamycin, the block in prApe1 processing and GFP-Atg8 cleavage was partially overcome, presumably because Atg17, which also binds Atg9, can compensate for the loss of the ability of Atg11 to bind to Atg9 (34, 36). Additionally, our Pho8Delta60 assay shows that the interaction between Atg11 and Atg9 is not required for bulk autophagy and that mutations of the PLF motifs do not affect the other functions of Atg9.

Atg11 is not the only binding partner of Atg9-NTD, Atg17 and Atg13 also interact with this domain (19, 33, 34). The capacity of Atg9-NTD to engage in multiple interactions is in line with its disordered nature. Indeed, IDPs through the fast sampling of a large conformational space are able to form complexes with different ligands and to act as interacting hubs in highly regulated processes (37).

This work set the base for future characterization on the binding properties of Atg9-NTD in order to understand the interplay between the different binding partners of Atg9 during the formation of the phagophore and the assembly of the Atg1 complex in both selective and bulk autophagy.

## Experimental procedures

### Protein expression and purification

A list of constructs can be found in Table S1. Full-length yeast Atg11 was expressed and purified as described previously (6). For the NMR titration, Atg11 was dialyzed against 20 mM Bis-Tris pH 6, 300 mM NaCl. Atg9-NTD constructs were all expressed in *E. coli* Rosetta pLysS cells in minimal medium containing ^15^N-labeled ammonium chloride and ^13^C-glucose as sole nitrogen and carbon sources, respectively. Atg9-NTD expression was induced at an *A*_600nm_ of 0.6 by addition of 0.2 mM IPTG. The cells were collected after 16 h of expression at 18 °C by centrifugation at 6000 rpm for 10 min and resuspended in 30 ml per liter of bacterial culture of ice-cold lysis buffer containing 20 mM Tris pH 7.4, 300 mM NaCl 10 mM Imidazole, 2 mM MgCl_2_, 2 mM β−mercaptoethanol, Roche complete protease inhibitors, and Benzonase (Sigma). Bacteria were lysed by passing through a French press, the cell lysate was then cleared by centrifugation at 20,000 rpm for 20 min. The supernatant containing the soluble protein fraction was loaded onto a HiTrap 5 ml affinity column (GE Healthcare) and eluted through an imidazole gradient. Protein-containing fractions were pooled, concentrated, applied onto a Superdex 200 column (16/600 prep grade, GE Healthcare), and eluted with a buffer containing 20 mM Bis-Tris pH 6, 300 mM NaCl for NMR experiments or 20 mM Tris pH 7.4, and 300 mM NaCl for interaction assays (MBPPI and ITC). For sample destined to resonance assignment, the EGFP tag was cleaved prior to the size-exclusion step through incubation overnight at 4 °C with 3C protease (1 mg of protease per 50 mg of protein).

### NMR spectroscopy

For the resonance assignment, the sample consisted of 0.4 mM of uniformly ^15^N, ^13^C labeled Atg9-NTD (1–285). All spectra were acquired at 293 K on a Bruker AVANCE III HD 800 MHz spectrometer using the 5 mm TCI-HCN cryo-probe. Assignment of ^1^H, ^13^C, and ^15^N backbone and (partial) side-chain resonances was achieved using three-dimensional (3D) HNCO experiment and a set of five-dimensional (5D) experiments: HN(CA)CONH (38), (HACA)CON(CA)CONH (39) and HabCabCONH (38). All experiments were performed using sparse nonuniform sampling of indirectly detected time domains in order to increase resolution. The 3D HNCO experiment was used as a base spectrum for SMFT (sparse multidimensional Fourier transform) processing of higher-dimensionality experiments (38). Sampling artifacts from all experiments were removed using SSA (signal separation algorithm (40)), implemented into “cleaner” program, available from http://nmr.cent3.uw.edu.pl/software. Detailed acquisition parameters are listed in Table S2.

The resonance assignment was performed using the TSAR program (41). The input data for TSAR were prepared using Sparky software. Chemical shifts were deposited in the BMRB (42) under the accession number 51011.

For ^1^H-^15^N HSQC-based titrations, the sample consisted of 0.2 mM of Atg9-NTD (29–255)-mCherry to which Atg11 was added stepwise from a 0.1 mM solution of EGFP-Atg11. All spectra were acquired at 298 K on a Bruker AVANCE spectrometers operating at 600 MHz. All spectra were processed using NMRPipe/NMRDraw (43) and analyzed with NMRFAM Sparky (44).

### Microscopy-based protein–protein interaction assay

GFP trap beads were equilibrated with measurement buffer (20 mM Tris pH 7.4 and 300 mM NaCl) and incubated for 1 h with 10 μM of EGFP-Atg11. The beads were washed and incubated with 1 μM solution of Atg9-NTD in 20 mM pH 7.4 and 300 mM NaCl. The mixtures were incubated for at least 30 min at room temperature before imaging. Confocal images were acquired using a Zeiss LSM 700 with a Plan-Apochromat 20 × /0.8 objective. Quantification was done by drawing a line across each bead in Fiji. The intensity of the fluorescence along that line was integrated after background signal subtraction. The signal of the prey was normalized by the signal of the bait. Values were averaged for each condition within each replicate and then among replicates.

### Isothermal titration calorimetry

Measurements were performed with a TA instrument Nano ITC microcalorimeter. Experiments were carried out at 25 °C in 20 mm Tris, pH 7.4, 300 mM NaCl. The reference cell contained Milli-Q water. The concentration of EGFP-Atg11 in the reaction cell was 50 to 100 μM. The concentration of Atg9-NTD in the syringe was 300 to 500 μM. The titration consisted of 19 successive injections of 4 μl, with a stirring speed of 350 rpm, separated by intervals of 300 s. The heat release induced by the injection of buffer in Atg11 was subtracted from the raw data before analysis (Fig. S4). Data analysis was done assuming a single binding site. Two replicates were used to calculate average values as well as standard deviations (Fig. S4).

### Yeast strains and manipulation

Yeast strains are listed in Table S3. All experiments were performed with BY4741 yeast strains unless stated otherwise. Genetic modifications were done by PCR and/or homologous recombination using standard techniques. Multiple deletions were generated by mating and dissection. Plasmid DNA was transformed to yeast according to a LiOAc/ssDNA/PEG transformation protocol.

### Ape1 processing assay

A yeast strain lacking Atg9 was transformed with plasmids expressing Atg9-TAP or mutations thereof, grown to mid-log phase in synthetic selection medium (SD: 0.17% yeast nitrogen base, 0.5% ammonium sulfate, 2% glucose, amino acids as required), and where indicated, treated with 220 nM rapamycin for 4 h at 30 °C. Cultures were precipitated with 7% trichloroacetic acid (TCA), pellets were washed with acetone, dried, and resuspended in urea loading buffer (120 mM Tris-HCl pH 6.8, 5% glycerol, 8 M urea, 143 mM beta-mercaptoethanol, 8% SDS). The samples were analyzed by immunoblotting using rabbit anti-CBP antibody kindly provided by C. Ungermann and rabbit anti-Ape1 antiserum kindly provided by C. Kraft. Results from three independent experiments were quantified using Image Lab (Bio-Rad).

### GFP-Atg8 cleavage assay

A yeast strain lacking Atg9 was transformed with a GFP-Atg8 expressing plasmid and plasmids expressing Atg9-TAP or mutations thereof, grown to mid-log phase in synthetic selection medium (SD: 0.17% yeast nitrogen base, 0.5% ammonium sulfate, 2% glucose, amino acids as required), and where indicated, treated with 220 nM rapamycin for 4 h at 30 °C. Whole cell lysates were prepared by TCA extraction (described above) and analyzed by immunoblotting using rabbit anti-CBP antibody kindly provided by C. Ungermann and a mouse anti-GFP antibody (Max Perutz Labs, Monoclonal antibody facility).

### Pho8Delta60 assay

Yeast cells were grown to mid-log phase in YPD (1% yeast extract, 2% peptone, 2% glucose) and starved for 4 h in nitrogen starvation medium (SD-N: 0.17% yeast nitrogen base without amino acids, 2% glucose).

In total, 50 OD600 units of yeast culture were harvested by centrifugation. The pellets were washed with dH2O and ice-cold 0.85% NaCl containing 1 mM PMSF and resuspended in 8 μl/OD600 unit lysis buffer [20 mM PIPES pH 6.8, 0.5% Triton X-100, 50 mM KCl, 100 mM potassium acetate, 10 mM MgSO4, 10 μM ZnSO4, 1 mM PMSF, cOmplete protease inhibitor cocktail (Roche)]. Cells were lysed by bead beating, and extracts were cleared by centrifugation. Protein concentration of the supernatant was adjusted to 50 μg in 100 μl lysis buffer. In total, 400 μl reaction buffer (0.4% Triton X-100, 10 mM MgSO4, 10 μM ZnSO4, and 250 mM Tris-HCl pH 8.5) containing 6.25 mM α-naphthylphosphate (Sigma-Aldrich) was added to enzymatic reactions, or only reaction buffer was added to control reactions. Reactions were incubated at 37 °C for 10 min and stopped by adding 500 μl stop buffer (1 M glycine pH 11). A 405 was measured using a plate reader. A standard curve was generated by using a dilution series of the product (1-naphtol, Sigma-Aldrich).

Three independent replicates were performed and activity was calculated the following: activity = [pNPP in nmol]/(t[min]∗[protein in mg]).

### Coimmunoprecipitation

*S. cerevisiae* Atg9-GFP Atg11Δ, Atg9-M1+2-GFP Atg11Δ, and Atg9Δ Atg11Δ cells were transformed with pRS316-mCh-Atg11 or empty pRS316 plasmid using the LiAc/SS carrier DNA/PEG method. Cells were grown to mid-log phase in YPD, harvested by centrifugation, and resuspended in IP buffer (25 mM Tris-HCl pH 7.4, 150 mM NaCl, 0.2% NP-40, 1 mM PMSF, Protease inhibitor cocktail [Roche], Protease inhibitor mix FY [Serva]). Droplets were frozen in liquid nitrogen and milled in a cryogenic grinder (SPEX Freezer mill). Yeast powder was resuspended in IP buffer and cleared by centrifugation at 5000*g* for 5 min at 4 °C. Lysates were incubated with GFP-trap magnetic agarose beads (Chromotek) o/n at 4 °C on a turning wheel. Beads were washed three times in IP buffer, resuspended in urea loading buffer (116 mM Tris-HCl pH 6.8, 4.9% glycerol, 8 M Urea, 8% SDS), and boiled for 10 min at 60 °C. Samples were analyzed by Western blot using mouse anti-Atg11 and mouse anti-GFP antibodies (Max Perutz Labs antibody facility), and bands were quantified using the BioRad Image Lab program.

### Quantitative live cell imaging

Plasmid DNA was transformed to yeast in stationary phase grown on YPD-agar according to a LiOAc/ssDNA/PEG transformation protocol. Transformed yeast strains were grown in synthetic defined minimal medium (SD; 1.7 g/l yeast nitrogen base without amino acids and ammonium sulfate (Formedium), 5 g/l ammonium sulfate, 2 g/l glucose) supplemented with the appropriate amino acid drop-out mix (CSM; Formedium) to log phase for imaging. Cells were immobilized with concavalinA (Sigma). Widefield images were obtained on a Deltavision ULTRA Epifluorescence Microscope using a UPlanSApo 100 × /1.4 Oil objective. Images were analyzed using the Fiji software. For the quantification, approximately 1000 cells were counted per construct (three experiments each). The plot was generated with GraphPad Prism.

## Data availability

Chemical shifts were deposited in the BMRB under the accession number 51011.

## Supporting information

This article contains supporting information (45).

## Conflict of interest

Sascha Martens is member of the scientific advisory board of Casma Therapeutics.
