# Supplementary material for: Mechanism of Atg9 recruitment by Atg11 in the cytoplasm-to-vacuole targeting pathway
Source: J Biol Chem. 2022 Jan 8;298(2):101573. doi: 10.1016/j.jbc.2022.101573 (PMC8814668; doi:10.1016/j.jbc.2022.101573)
Supplement: Supplemental Figures S1–S4 and Tables S1–S3 [file mmc1.pdf]

Supporting information for

Mechanism of Atg9 recruitment by Atg11 in the cytoplasm-to-vacuole targeting pathway

Nicolas Coudevylle[1,*], Bartłomiej Banaś[2], Verena Baumann[1], Martina Schuschnig[1], Anna Zawadzka-Kazimierczuk[2], Wiktor Koźmiński[2] and Sascha Martens[1,*].

[1]Max Perutz Laboratories, University of Vienna, Dr.-Bohrgasse 9, 1030 Vienna, Austria

[2]Biological and Chemical Research Centre, Faculty of Chemistry, University of Warsaw, Żwirki i Wigury 101, 02-089 Warsaw, Poland

Supplementary figure 1: Neighbour corrected structural propensity {Tamiola, 2010 #540} of Atg9-NTD based on 1H, 15N, and 13C experimental chemical shifts.

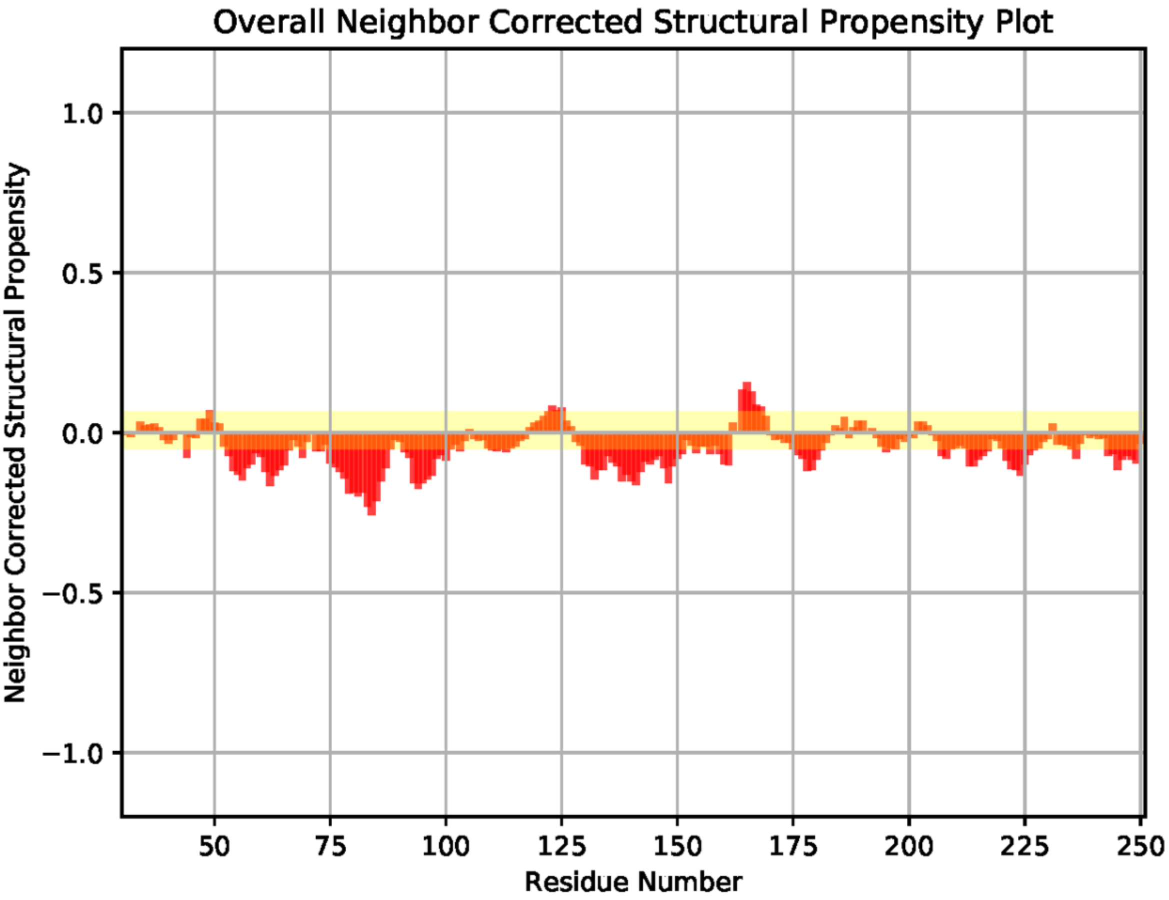

Supplementary figure 2: Overlay of the 1H-15N HSQC spectra of Atg9-NTD segment 1-285 (red resonances) and 29-255 (blue resonances).

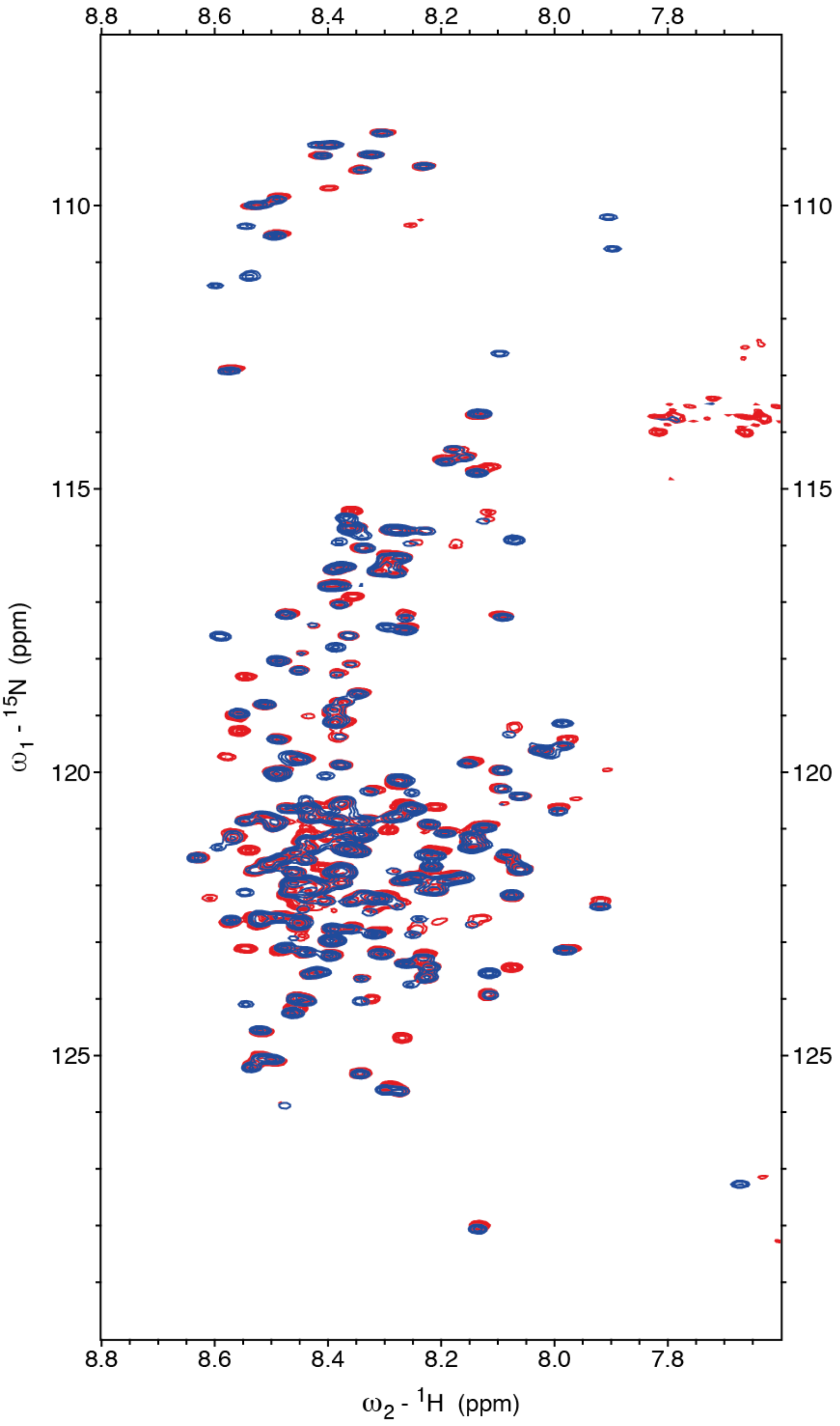

Table S1. Table of constructs

**Identification Number**	**Vector**	**Expression system**	**Expressing**	**Published**
SMC195	pRS316	yeast	-	
SMC327	pRS416	yeast	Atg9-TAP	Papinsky *et al.*, 2014
SMC1064	pFastBac HT	Sf9 cells	6xHis-TEV-mEGFP-Atg11	Sawa-Makarska *et al.*, 2020
SMC1340	pET-M11	*E. coli*	6xHis-TEV-Atg9(1-285)-3C-eGFP	This study
SMC1247	pET-Duet	*E. coli*	6xHis-Atg9(S19D)(1-255)-mCherry	This study
SMC1487	pET-Duet	*E. coli*	6xHis-Atg9(29-255)-mCherry	This study
SMC1510	pET-Duet	*E. coli*	6xHis-Atg9(S19D, L164A/F165A)(1-255)-mCherry	This study
SMC1511	pET-Duet	*E. coli*	6xHis-Atg9(S19D, L188A/F189A)(1-255)-mCherry	This study
SMC1512	pET-Duet	*E. coli*	6xHis-Atg9(S19D, L164A/F165A/L188A/F189A)(1-255)-mCherry	This study
SMC1574	pRS416	yeast	Atg9-TAP L164A/F165A	This study
SMC1575	pRS416	yeast	Atg9-TAP L188A/F189A	This study
SMC1576	pRS416	yeast	Atg9-TAP L164A/F165A/L188A/F189A	This study
SMC199	pRS315	yeast	GFP-Atg8	Kraft et al., 2012

Table S2. Parameters of the multidimensional NMR experiments used for the assignment of Atg9-NTD(1-285). (nucl: nucleus; indir dim: indirect dimension, sw: spectral width, t_{max}: maximum evolution time, ni: number of non-uniform sampling complex points).

Experiment	Experimental parameters													
	indir dim1			indir dim2			indir dim3			indir dim4			ni	exp time (h)
	nucl	sw (Hz)	t_{max} (ms)	nucl	sw (Hz)	t_{max} (ms)	nucl	sw (Hz)	t_{max} (ms)	nucl	sw (Hz)	t_{max} (ms)		
3D HNCO	C’	2500	50	N	2300	76	-	-	-	-	-	-	2500	16
5D HN(CA)CONH	H^N	2500	10	N	2300	27	C’	2500	27	N	2300	27	2800	72
5D (HACA)CON(CA)CONH	C’	2500	27	N	4000	54	C’	2500	27	N	2300	27	5850	155
5D HabCabCONH	Hab	5600	11	Cab	13000	7	C’	2500	27	N	2300	27	2000	43

Table S3. Table of yeast strains.

Name	Genotype	Background	Reference
BY4741	his3Δ1 leu2Δ0 met15Δ0 ura3Δ0 MATa	-	Euroscarf
SMY056	*atg9::kan* MATa	BY4741	Euroscarf
